# Supplementary material for: The Lipophilic Purine Nucleoside—Tdp1 Inhibitor—Enhances DNA Damage Induced by Topotecan In Vitro and Potentiates the Antitumor Effect of Topotecan In Vivo
Source: Molecules. 2022 Dec 31;28(1):323. doi: 10.3390/molecules28010323 (PMC9822400; doi:10.3390/molecules28010323)
Supplement: Supplementary file 1 [file molecules-28-00323-s001.zip › molecules-2030489-supplementary.pdf]

The Lipophilic Purine Nucleoside—Tdp1 Inhibitor—Enhances DNA Damage Induced by Topotecan In Vitro and Potentiates the Antitumor Effect of Topotecan In Vivo

Irina A. Chernyshova [1], Aleksandra L. Zakharenko [1], Nikolay N. Kurochkin [2], Nadezhda S. Dyrkheeva [1], Tatyana E. Kornienko [1], Nelly A. Popova [3,4], Valeriy P. Nikolin [3], Ekaterina S. Ilina [1,4], Timofey D. Zharkov [1], Maxim S. Kupryushkin [1], Vladimir E. Oslovsky [2], Mikhail S. Drenichev [2] and Olga I. Lavrik [1,4,*]

1 Institute of Chemical Biology and Fundamental Medicine, Siberian Branch of the Russian Academy of Sciences, 630090 Novosibirsk, Russia
2 Engelhardt Institute of Molecular Biology, Russian Academy of Sciences, 119991 Moscow, Russia
3 Federal Research Centre Institute of Cytology and Genetics, Siberian Branch of the Russian Academy of Sciences, 630090 Novosibirsk, Russia
4 V. Zelman Institute for the Medicine and Psychology, Novosibirsk State University, 630090 Novosibirsk, Russia
* Correspondence: lavrik@niboch.nsc.ru

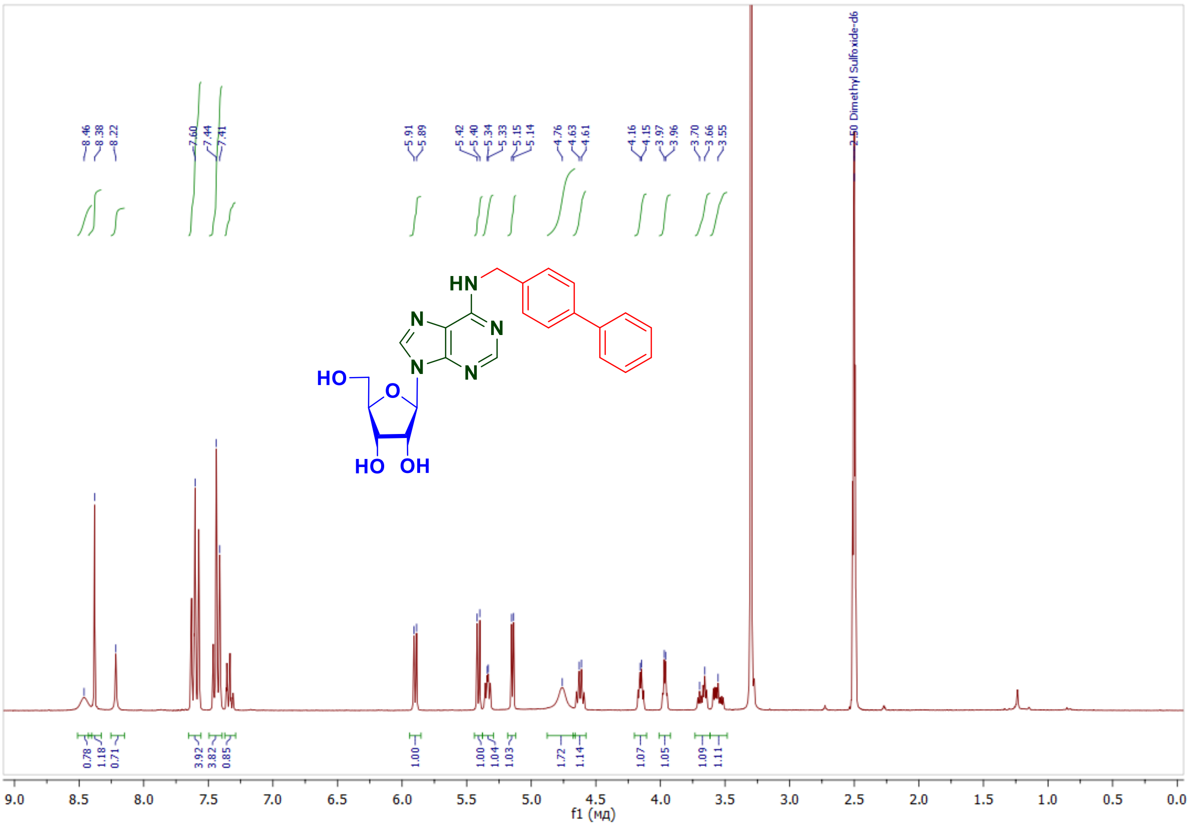

Figure S1A. ^{1}H-NMR spectrum (300.1 MHz) of N^6-biphenyl-4-methyladenosine (**5a**) in DMSO-d_6 at 298 K

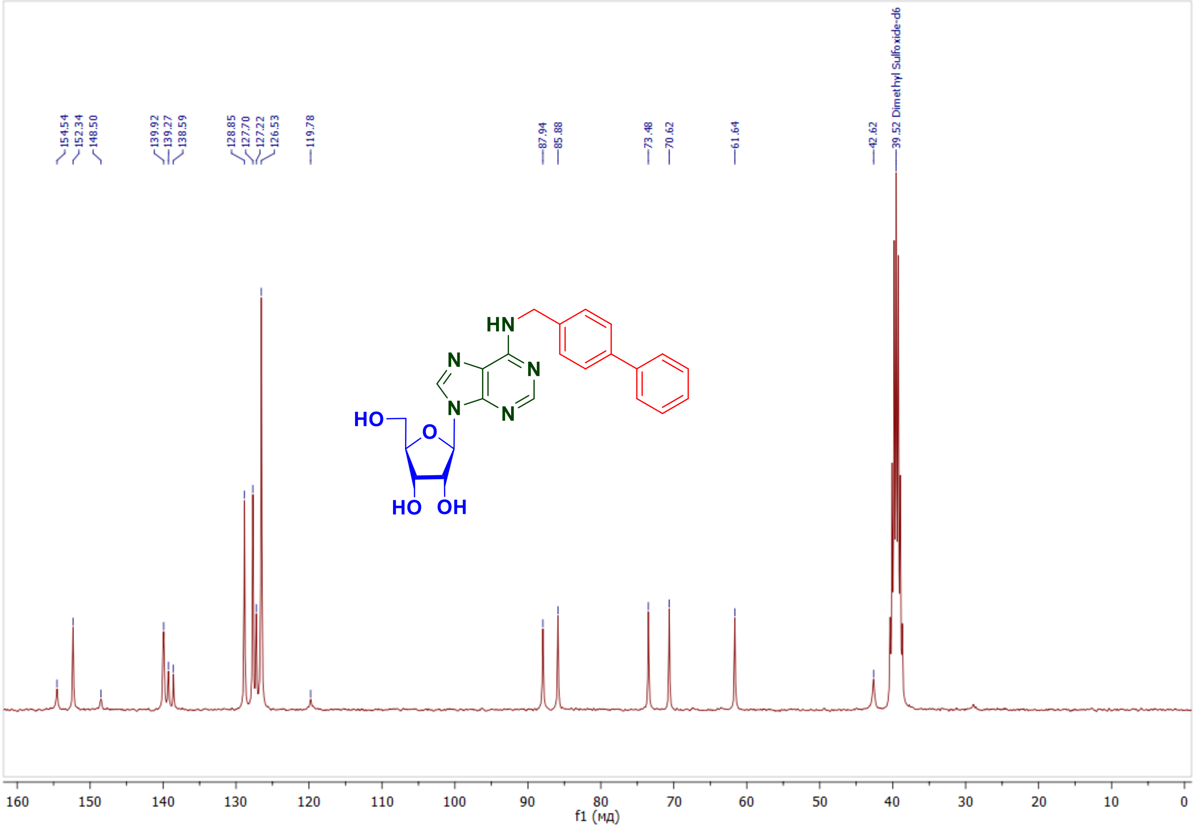

Figure S1B. ^{13}C-NMR spectrum (75.5 MHz) of *N*^{6}-biphenyl-4-methyladenosine (**5a**) in DMSO-d_6 at 298 K

Acquisition Parameter

Source Type	ESI	Ion Polarity	Positive	Set Nebulizer	0.4 Bar
Focus	Active	Set Capillary	4500 V	Set Dry Heater	180 °C
Scan Begin	50 m/z	Set End Plate Offset	-500 V	Set Dry Gas	6.0 l/min
Scan End	3000 m/z	Set Charging Voltage	2000 V	Set Divert Valve	Source
		Set Corona	0 nA	Set APCI Heater	0 °C

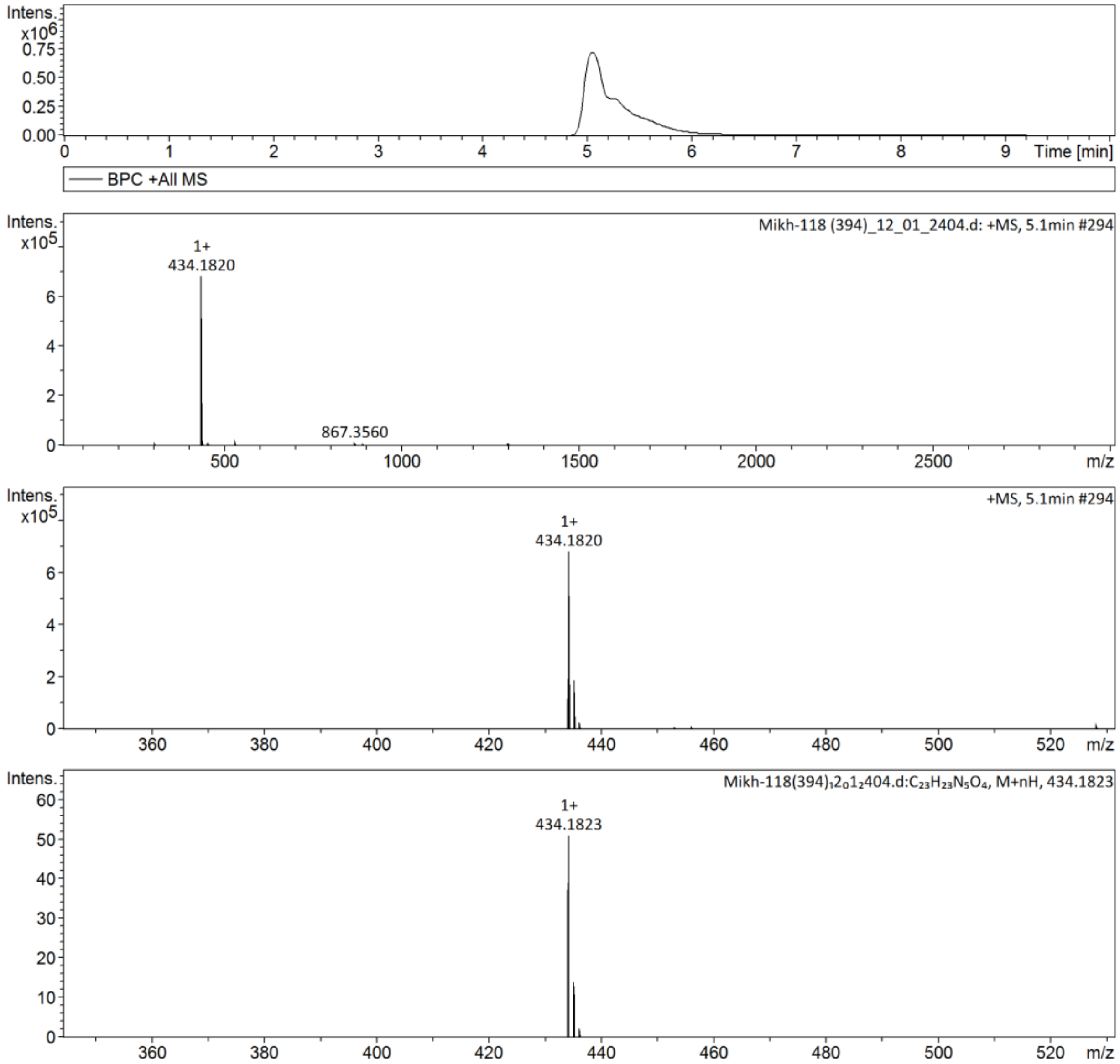

Figure S1C. HRMS High resolution mass spectrum (HRMS) of N^6-biphenyl-4-methyladenosine (**5a**)

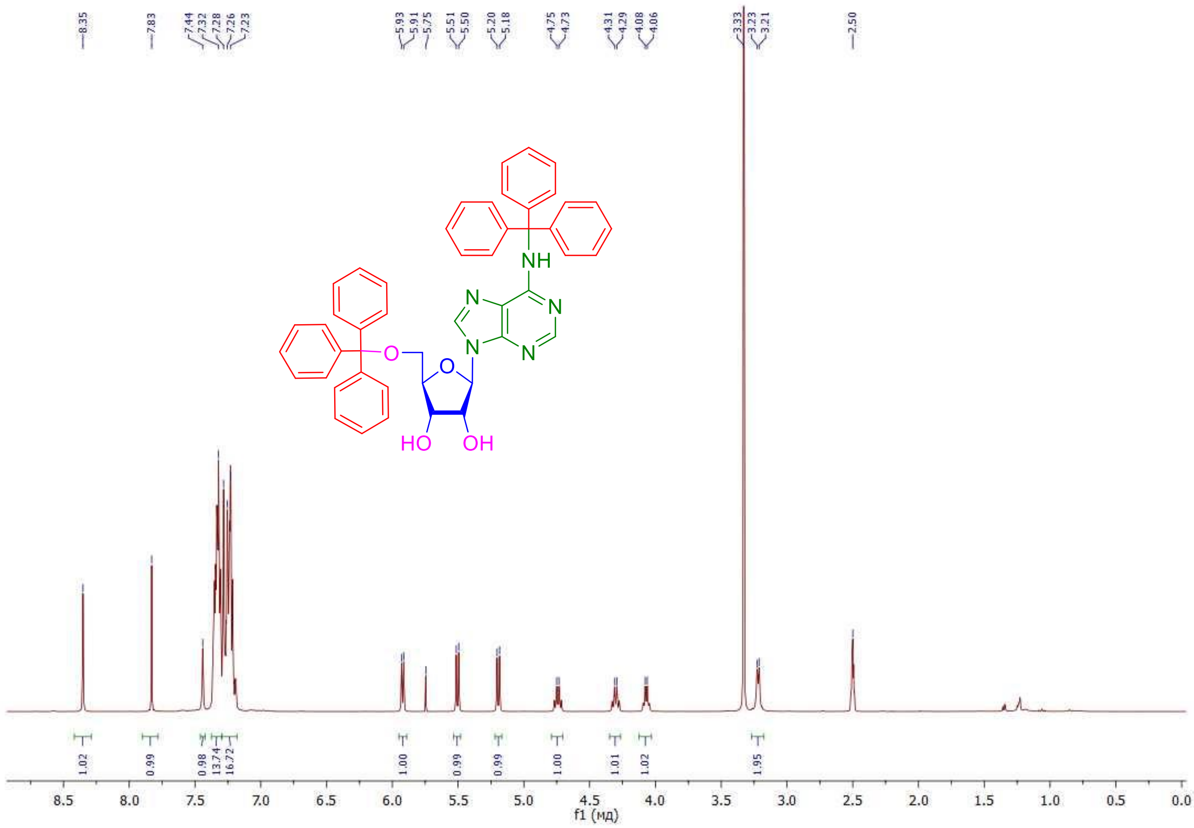

Figure S2. ^{1}H-NMR-spectrum (300 MHz) of 5′-*O*-trityl-*N*[6]-trityladenosine (**5f**) in DMSO-d_6 at 298 K.

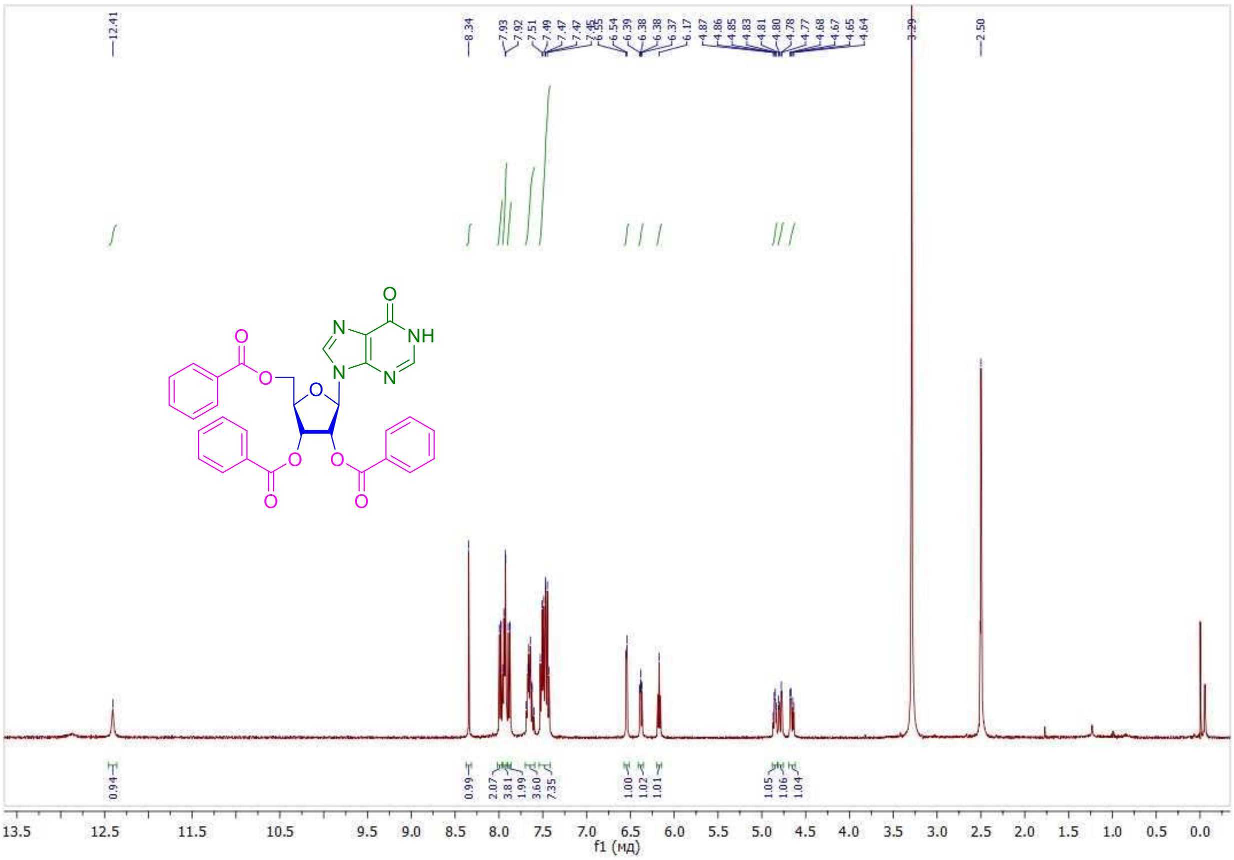

Figure S3. ^{1}H-NMR-spectrum (400 MHz) of 2′,3′,5′-tri-O-benzoylinosine (**6a**) in DMSO-d_6 at 298 K.

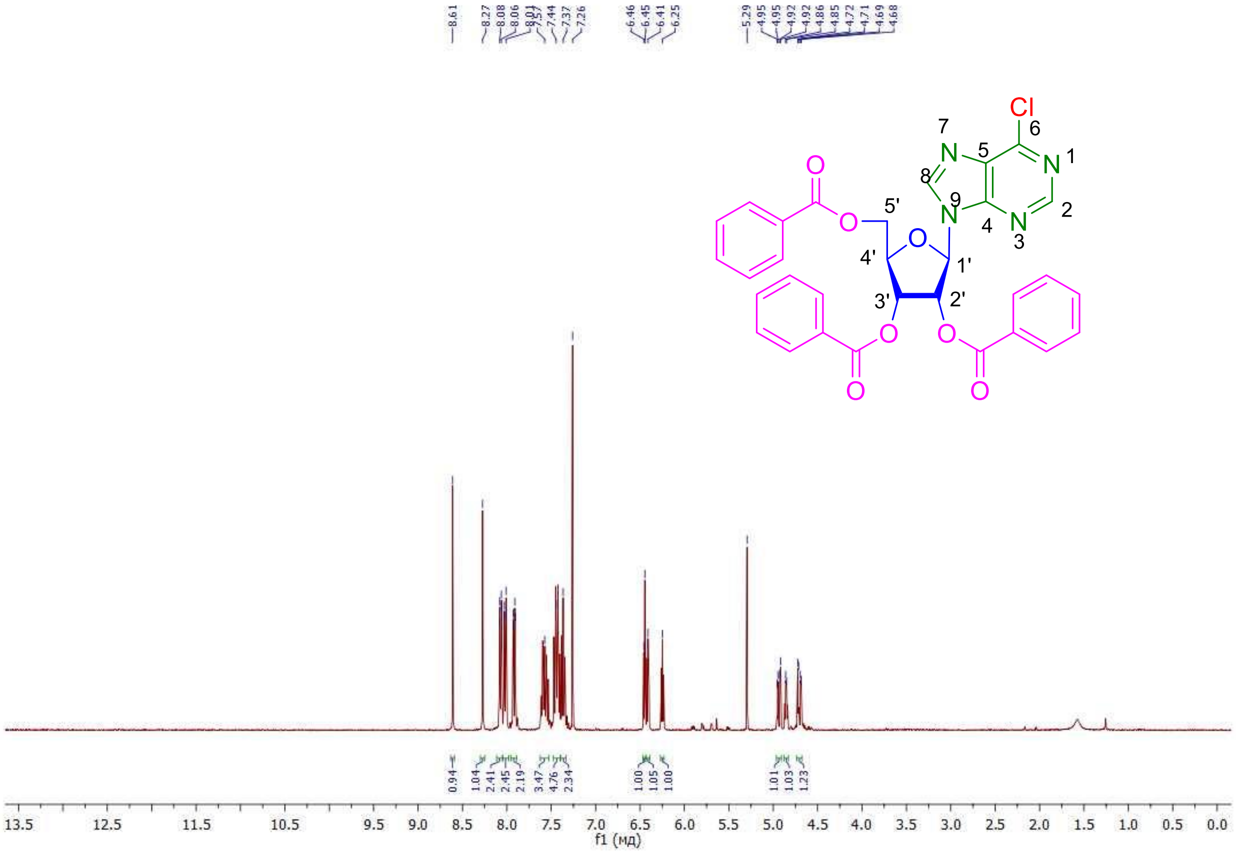

Figure S4A. ^{1}H-NMR-spectrum (400 MHz) of 6-Chloro-9-(2,3,5-tri-*O*-benzoyl-β-D-ribofuranosyl)purine (**6b**) in $CDCl_3$ at 298 K.

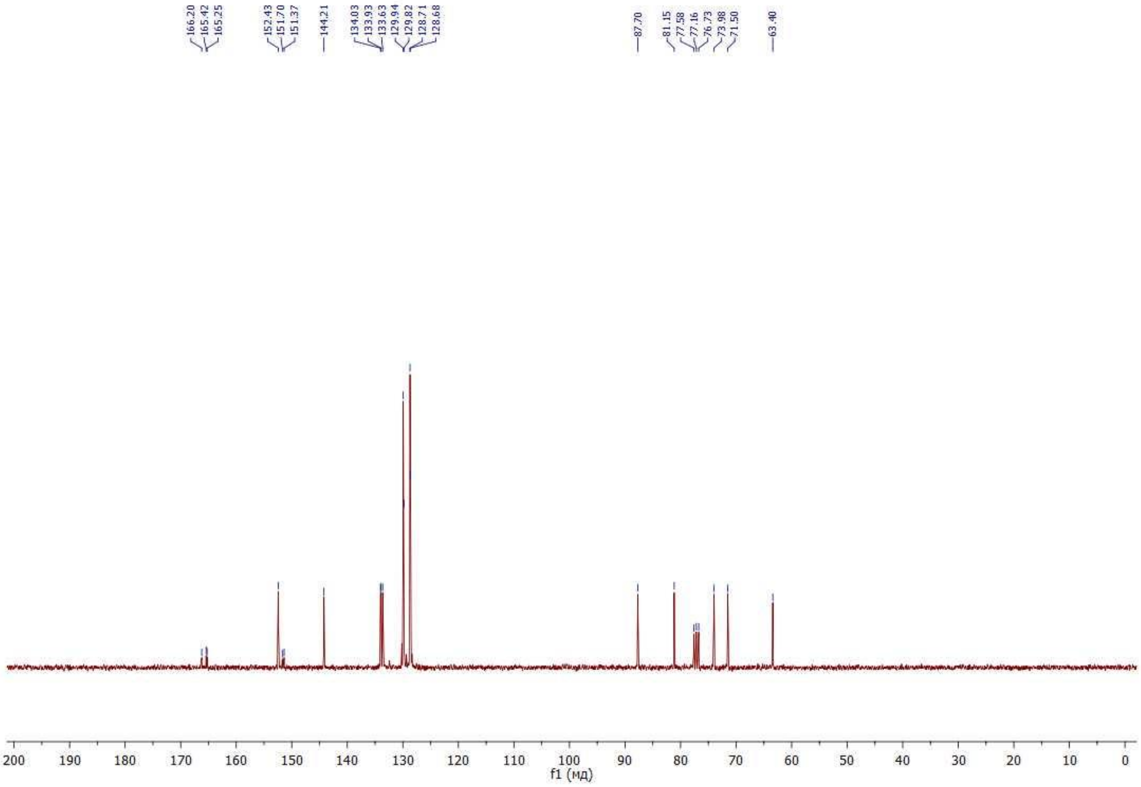

Figure S4B. ^{13}C-NMR-spectrum (75 MHz) of 6-Chloro-9-(2,3,5-tri-*O*-benzoyl-β-D-ribofuranosyl)purine (**6b**) in $CDCl_3$ at 298 K.

Display Report

Analysis Info

Analysis Name	D:\Data\Chizhov\IMB\Drenichev\sm4368_&clb.d	Acquisition Date	08.11.2022 16:47:22
Method	tune_wide.m	Operator	BDAL@DE
Sample Name	/CHIZ SM4368	Instrument / Ser#	maXis 43
Comment	CH3CN 100 %, dil. 2000, calibrant added.		

Acquisition Parameter

Source Type	ESI	Ion Polarity	Positive	Set Nebulizer	0.5 Bar
Focus	Active			Set Dry Heater	180 °C
Scan Begin	50 m/z	Set Capillary	4500 V	Set Dry Gas	4.0 l/min
Scan End	3000 m/z	Set End Plate Offset	-500 V	Set Divert Valve	Waste

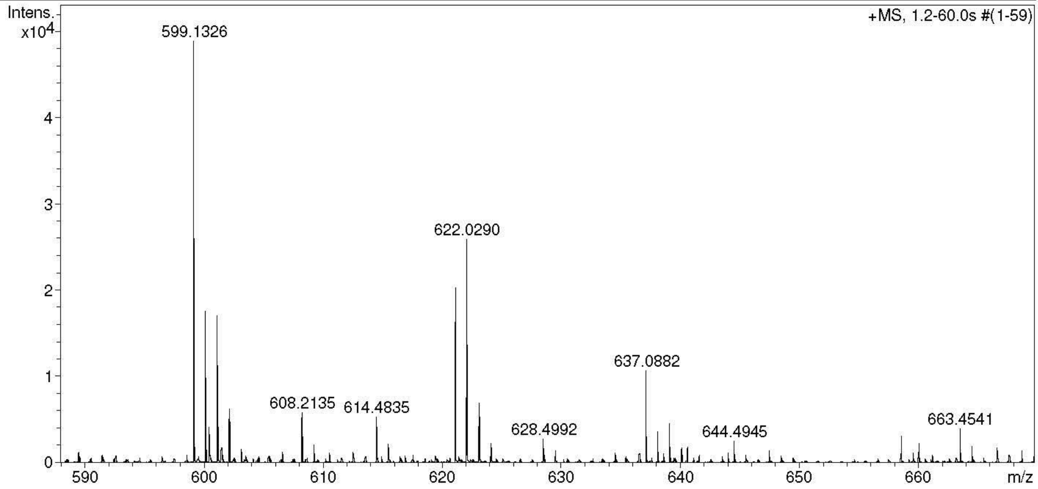

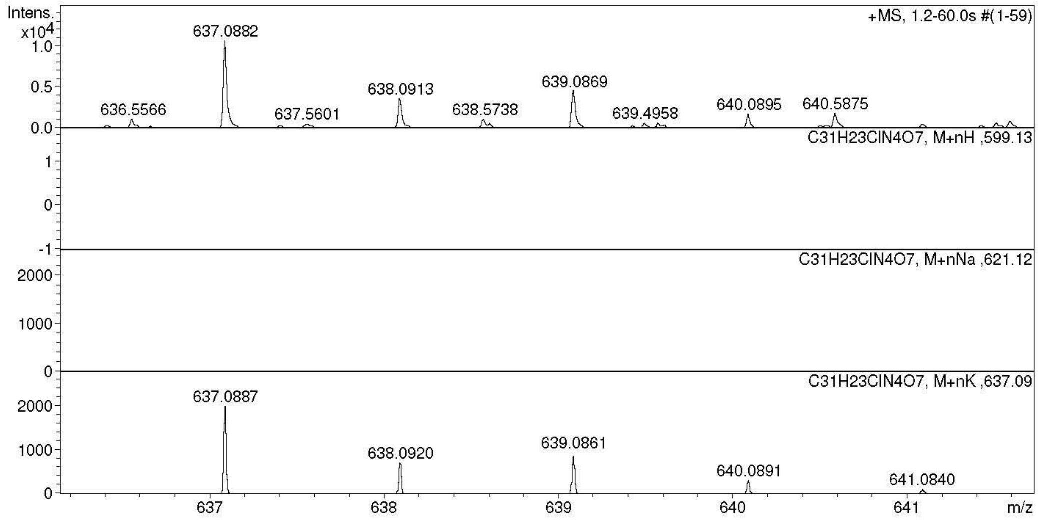

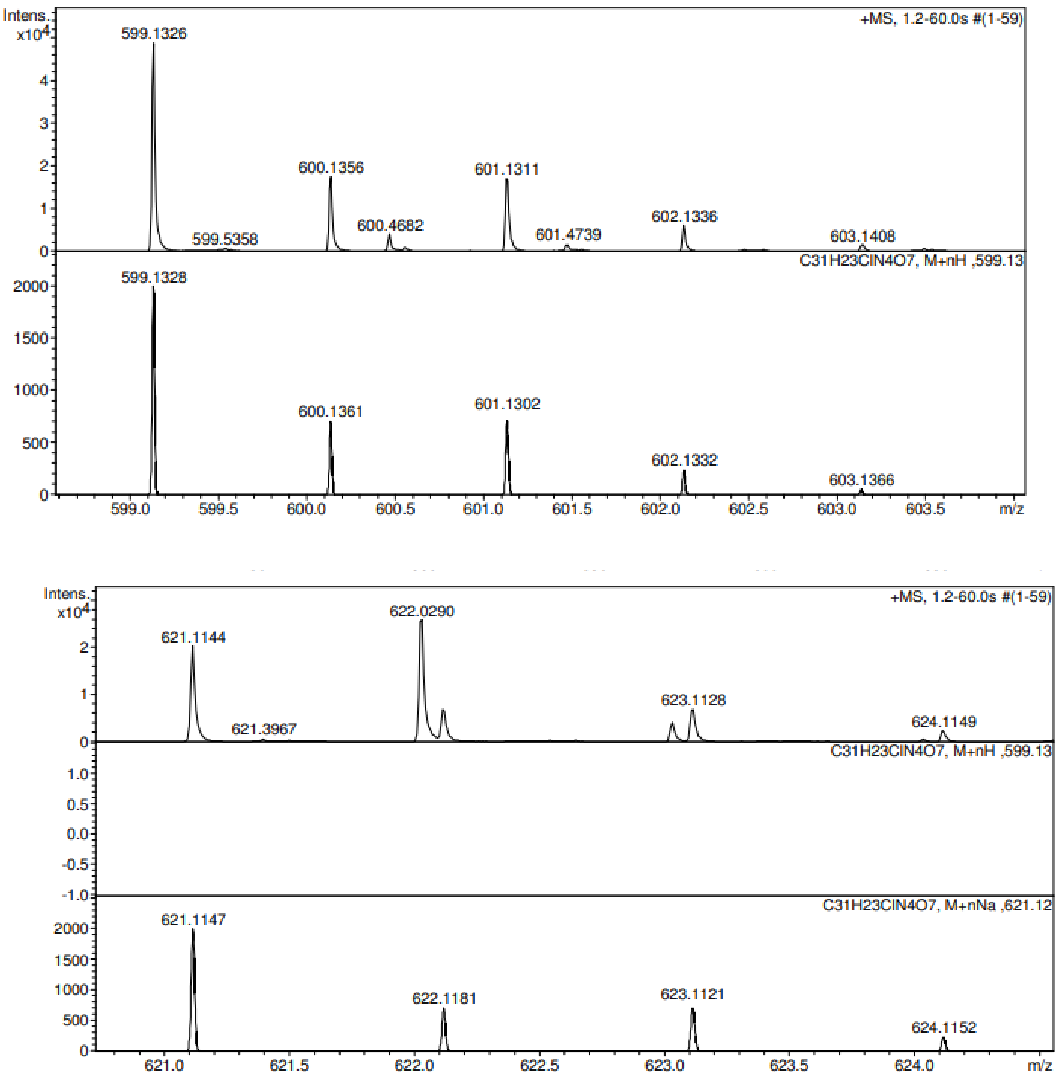

Figure S4C. HRMS analysis of of 6-Chloro-9-(2,3,5-tri-*O*-benzoyl-β-D-ribofuranosyl)purine (**6b**) (622.0290-calibrant peak).

m/z[$C_{31}H_{23}ClN_4O_7$+H^+] calculated 599.1328, found 599.1326, [$C_{31}H_{23}ClN_4O_7$+Na^+] calculated 621.1147, found 621.1144, [$C_{13}H_{23}ClN_4O_7$+K^+] calculated 637.0887, found 637.0882.

Residual peaks: [$C_{31}H_{23}ClN_4O_7$+MeCN+Na^++H^+] calculated 663.1491, found 663.4541, [$C_{31}H_{23}ClN_4O_7$+H_2O+2Na^++H^+] calculated 663.1236, found 663.4541, [$C_{31}H_{23}ClN_4O_7$+ 2MeCN+Na^+-N=CH-N] calculated 663.1617, found 663.4541, [$C_{31}H_{23}ClN_4O_7$+ H^+ 2H_2O+MeCN-CH] calculated 663.1727, found 663.4541, [$C_{31}H_{23}ClN_4O_7$+H^++2H_2O+MeCN-CH] calculated 663.1727, found 663.4541, [$C_{31}H_{23}ClN_4O_7$+2Na^+] calculated 644.1046, found 644.4945, [663.4541-Cl] calculated *628.4853*, found *628.4992*, [628.4992-N] calculated *614.4822*, found *614.4835*, [663.4541-CH=N] calculated 608.4245, found 608.2135.

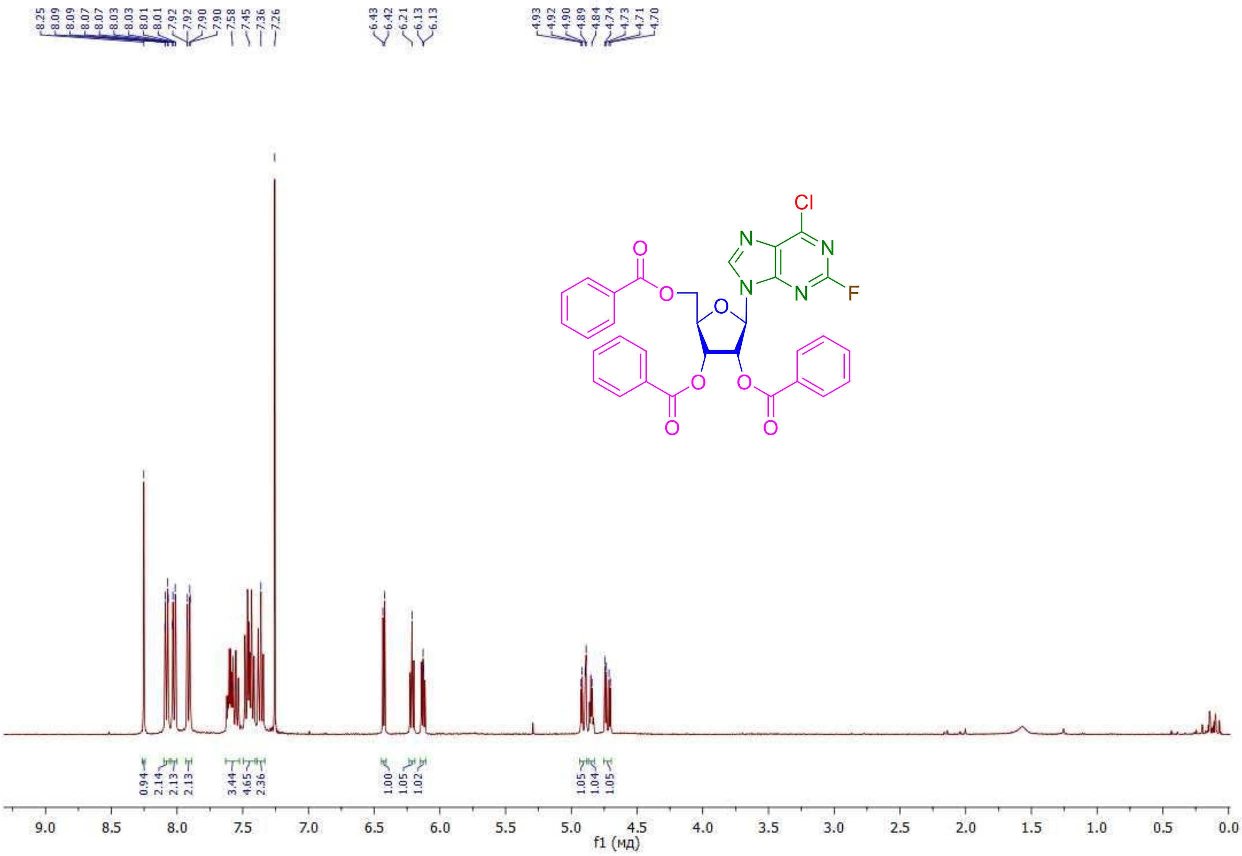

Figure S5A. ^{1}H-NMR-spectrum (400 MHz) of 2-Fluoro-6-chloro-9-(2,3,5-tri-*O*-benzoyl-β-D-ribofuranosyl)purine (**6c**) in $CDCl_3$ at 298 K.

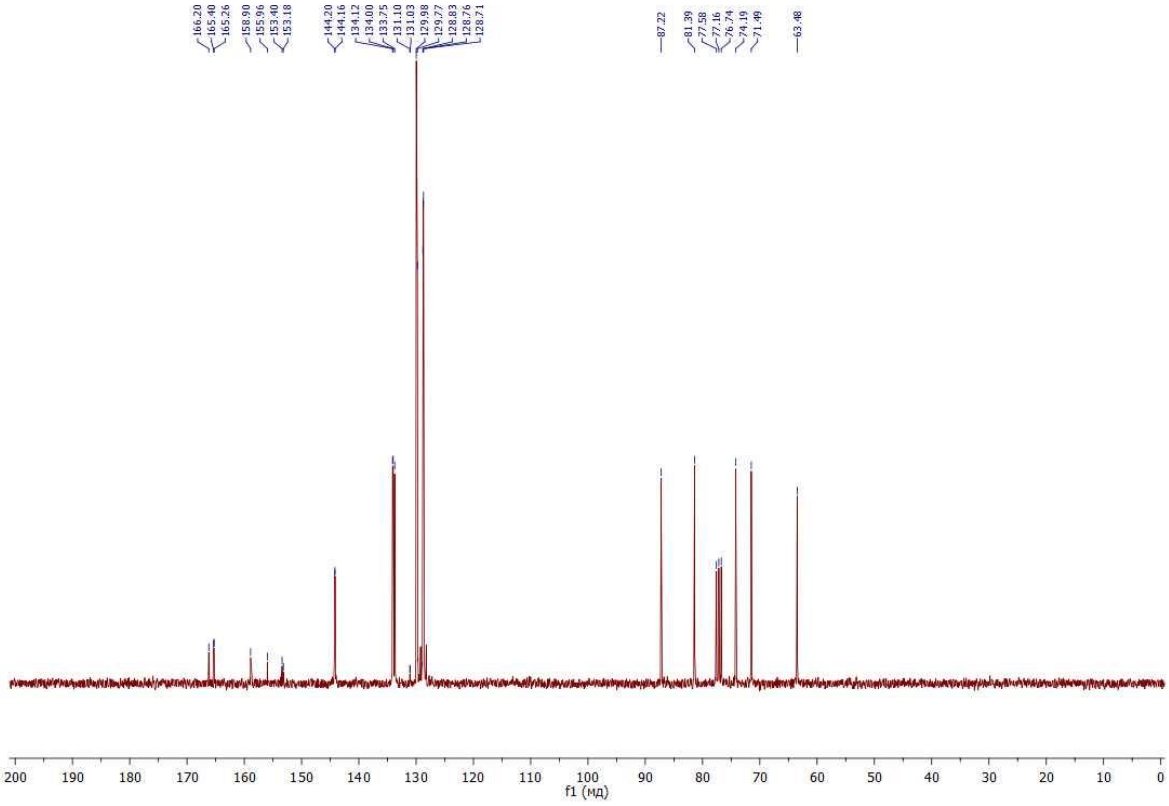

Figure S5B. ^{13}C-NMR-spectrum (75 MHz) of 2-Fluoro-6-chloro-9-(2,3,5-tri-*O*-benzoyl-β-D-ribofuranosyl)purine (**6c**) in $CDCl_3$ at 298 K.

Display Report

Analysis Info

Analysis Name	D:\Data\Chizhov\IMB\Drenichev\sm4370_&clb.d	Acquisition Date	08.11.2022 16:58:51
Method	tune_wide.m	Operator	BDAL@DE
Sample Name	/CHIZ SM4370	Instrument / Ser#	maXis 43
Comment	CH3CN 100 %, dil. 200, calibrant added.		

Acquisition Parameter

Source Type	ESI	Ion Polarity	Positive	Set Nebulizer	0.5 Bar
Focus	Active			Set Dry Heater	180 °C
Scan Begin	50 m/z	Set Capillary	4500 V	Set Dry Gas	4.0 l/min
Scan End	3000 m/z	Set End Plate Offset	-500 V	Set Divert Valve	Waste

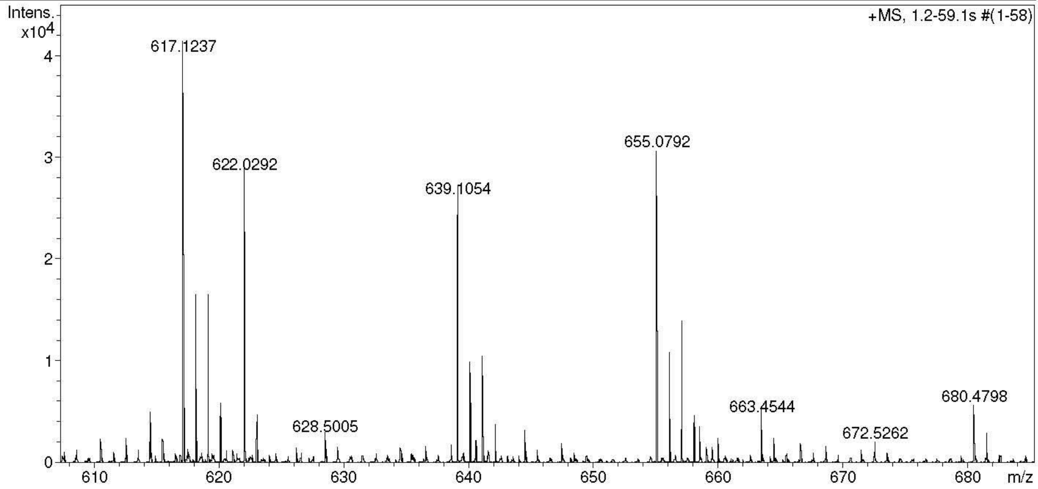

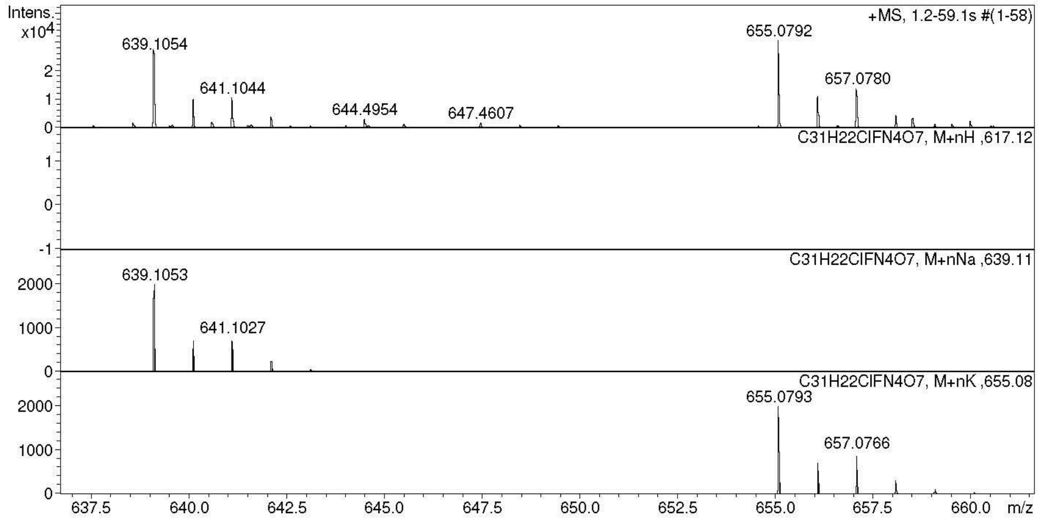

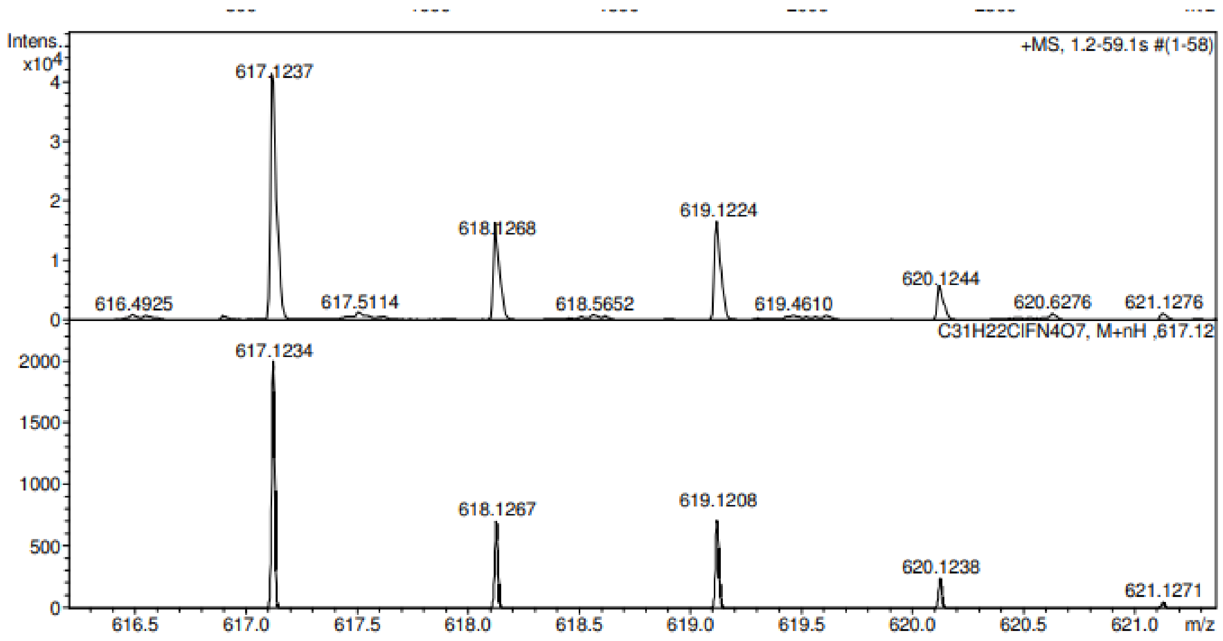

Figure S5C. HRMS analysis of 2-Fluoro-6-chloro-9-(2,3,5-tri-*O*-benzoyl-β-D-ribofuranosyl)purine (**6c**).

HRMS: *m/z*[$C_{31}H_{22}ClFN_4O_7$+H^+] calculated 617.1234, found 617.1237, [$C_{13}H_{22}ClFN_4O_7$+Na^+] calculated 639.1053, found 639.1054, [$C_{13}H_{22}ClFN_4O_7$+K^+] calculated 655.0793, found 655.0792.

Residual peaks: [$C_{31}H_{22}ClFN_4O_7$+MeCN+Na^+] calculated 680.1319, found 680.4798, [$C_{31}H_{22}ClFN_4O_7$+MeCN+Na^++H^+-F] calculated 663.1491, found 663.4544, [$C_{31}H_{22}ClFN_4O_7$+H_2O+2Na^++H^+-F] calculated 663.1236, found 663.4544, [$C_{31}H_{22}ClFN_4O_7$+ 2MeCN+Na^+-N=CH-N-F] calculated 663.1617, found 663.4544, [$C_{31}H_{22}ClFN_4O_7$+ H^+ 2H_2O+MeCN-CH-F] calculated 663.1727, found 663.4544, [$C_{31}H_{22}ClFN_4O_7$+H^++2H_2O+MeCN-CH-F] calculated 663.1727, found 663.4544, [663.4544-Cl] calculated *628.4856*, found *628.5005*, [628.4992-N] calculated *614.4835*, found *614.4822*, [663.4544-CH=N] calculated 608.4248, found 608.2135.

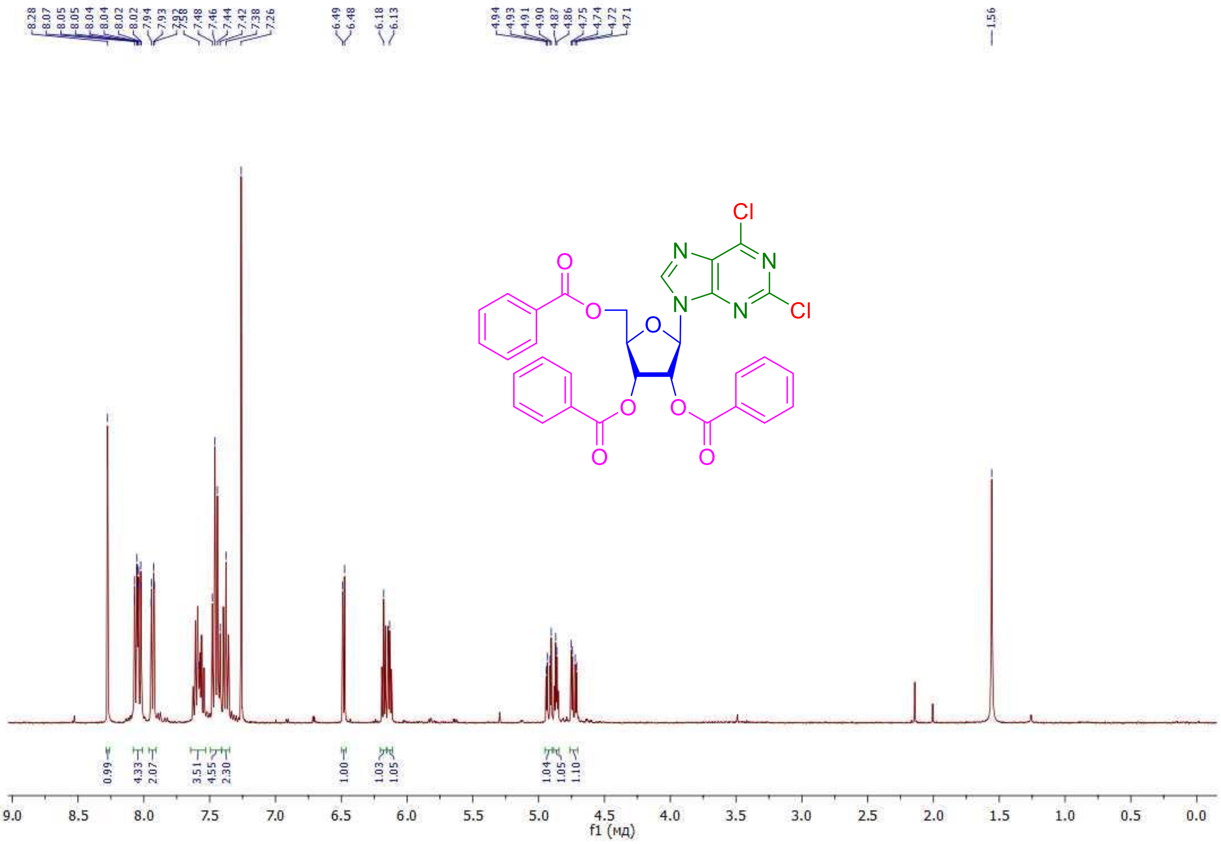

Figure S6A. ^{1}H-NMR-spectrum (400 MHz) of 2,6-dichloro-9-(2,3,5-tri-*O*-benzoyl-β-D-ribofuranosyl)purine (**6d**) in $CDCl_3$ at 298 K.

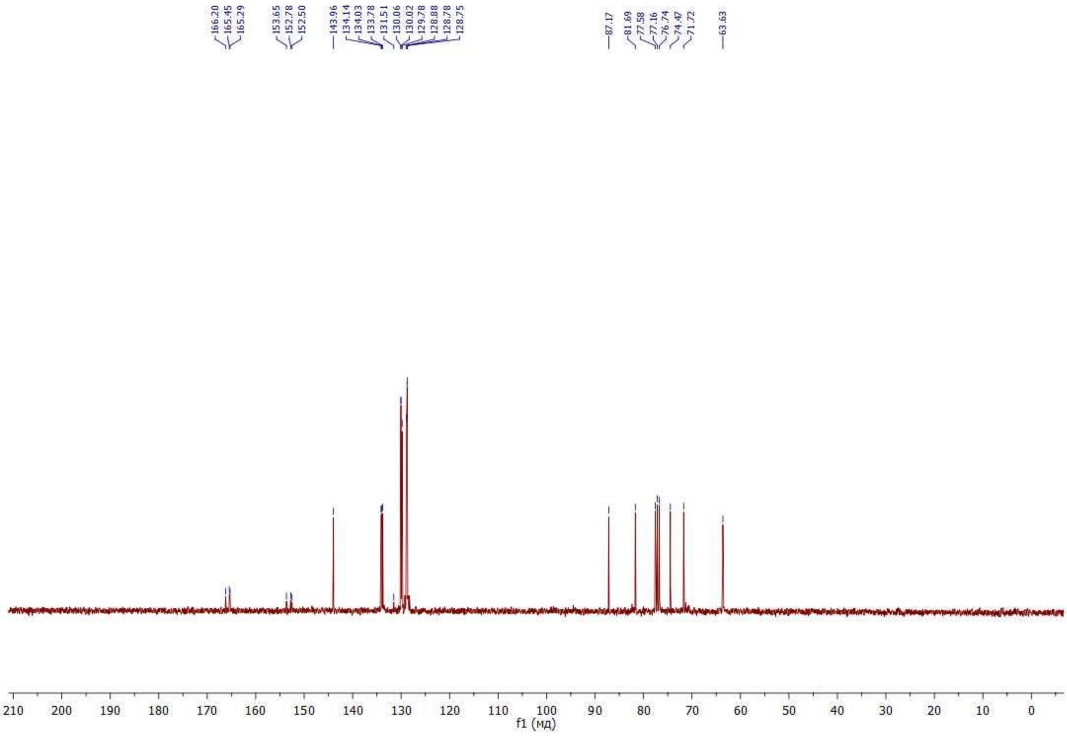

Figure S6B. ^{13}C-NMR-spectrum (75 MHz) of 2,6-dichloro-9-(2,3,5-tri-*O*-benzoyl-β-D-ribofuranosyl)purine (**6d**) in $CDCl_3$ at 298 K.

Display Report

Analysis Info

Analysis Name	D:\Data\Chizhov\IMB\Drenichev\sm4369_&clb.d	Acquisition Date	08.11.2022 16:53:04
Method	tune_wide.m	Operator	BDAL@DE
Sample Name	/CHIZ SM4369	Instrument / Ser#	maXis 43
Comment	CH3CN 100 %, dil. 2000, calibrant added.		

Acquisition Parameter

Source Type	ESI	Ion Polarity	Positive	Set Nebulizer	0.5 Bar
Focus	Active			Set Dry Heater	180 °C
Scan Begin	50 m/z	Set Capillary	4500 V	Set Dry Gas	4.0 l/min
Scan End	3000 m/z	Set End Plate Offset	-500 V	Set Divert Valve	Waste

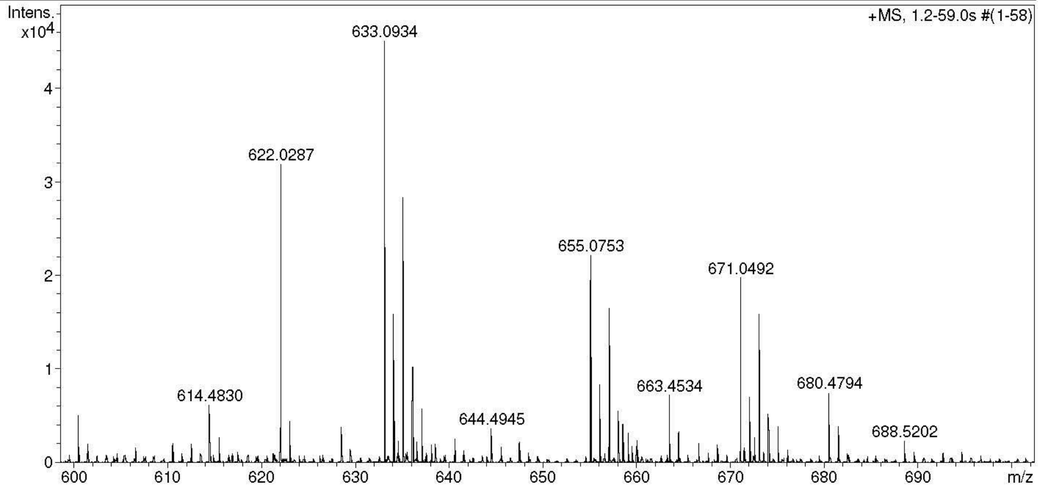

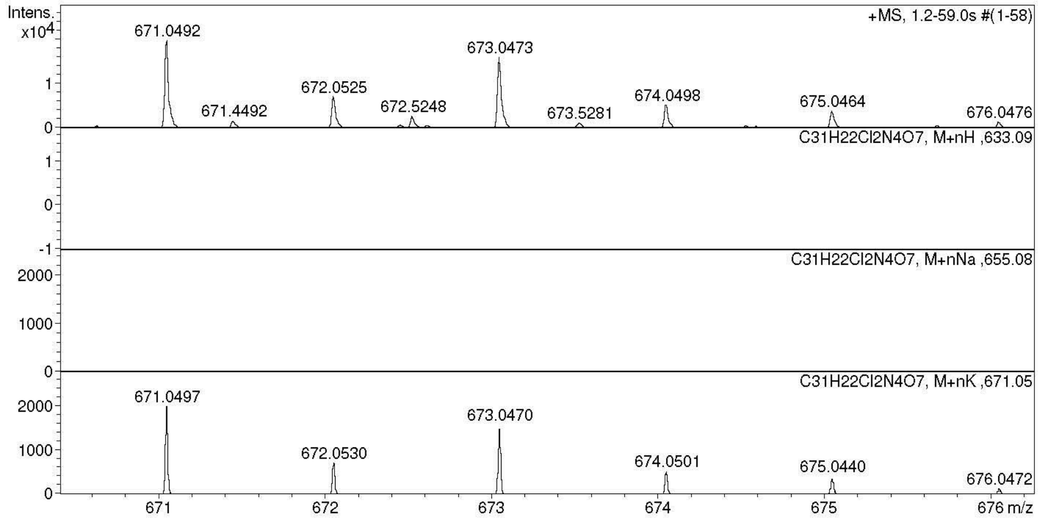

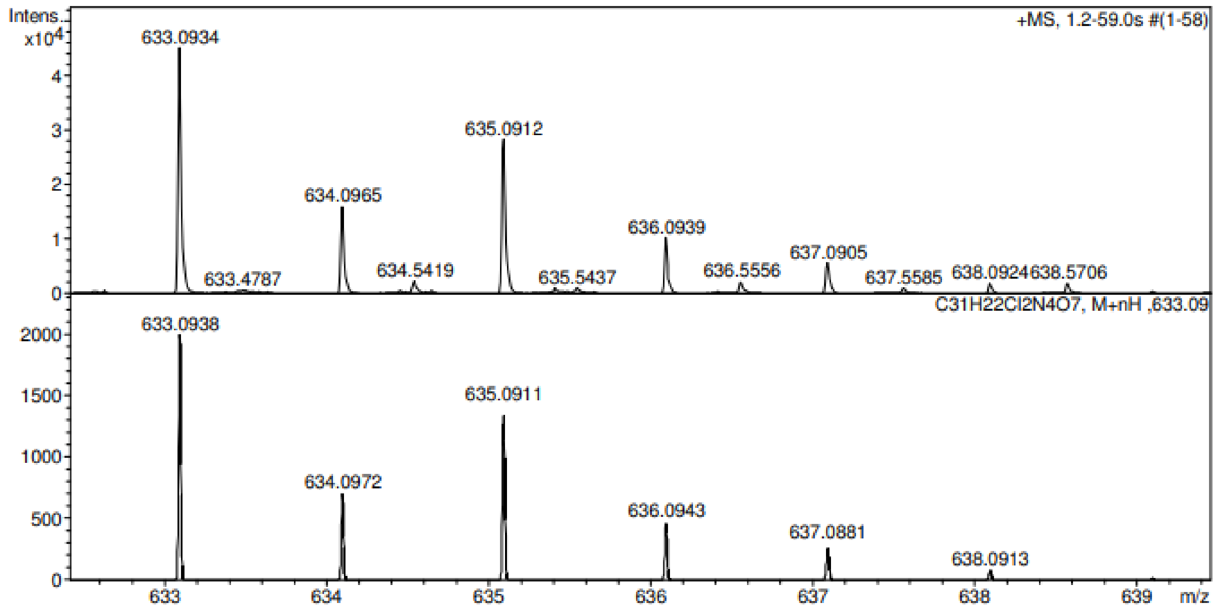

Figure S6C. HRMS analysis of 2,6-dichloro-9-(2,3,5-tri-*O*-benzoyl-β-D-ribofuranosyl)purine (**6d**).

HRMS: *m/z* [$C_{31}H_{22}Cl_2N_4O_7$+H^+] calculated 633.0938, found 633.0934, [$C_{13}H_{22}Cl_2N_4O_7$+Na^+] calculated 655.0758, found 655.0753, [$C_{13}H_{22}Cl_2N_4O_7$+K^+] calculated 671.0497, found 671.0492.

Residual peaks: [$C_{31}H_{22}Cl_2N_4O_7$+2MeCN+H^+-Cl] calculated 680.1780, found 680.4794, [$C_{31}H_{22}Cl_2N_4O_7$+MeCN+Na^++H^+-Cl] calculated 663.1491, found 663.4534, [$C_{31}H_{22}Cl_2N_4O_7$+H_2O+2Na^++H^+-Cl] calculated 663.1236, found 663.4534, [$C_{31}H_{22}Cl_2N_4O_7$+ 2MeCN+Na^+-N=CH-N-Cl] calculated 663.1617, found 663.4534, [$C_{31}H_{22}Cl_2N_4O_7$+ H^+ 2H_2O+MeCN-CH-Cl] calculated 663.1727, found 663.4534, [$C_{31}H_{22}Cl_2N_4O_7$+H^++2H_2O+MeCN-CH-Cl] calculated 663.1727, found 663.4534, [$C_{31}H_{22}Cl_2N_4O_7$+H^++2Na^+-Cl] calculated 644.1046, found 644.4945, [663.4534-Cl-N] calculated *614.4815*, found *614.4830*.

Table S1. Residual activities of PARP1 and PARP2, %, in the presence of 1 mM compounds

№	PARP1	PARP2
**1**	86±5	91±5
**2**	102±11	87±18
**3**	94±9	95±9
**4**	84±12	79±15
**5a**	112±11	101±24
**5b**	99±8	107±9
**5c**	92±14	104±16
**5d**	88±21	100±15
**5f**	100±10	113±18
**6a**	87±10	96±30
**6b**	86±8	75±16
**6c**	82±17	80±20
**6d**	96±12	86±9

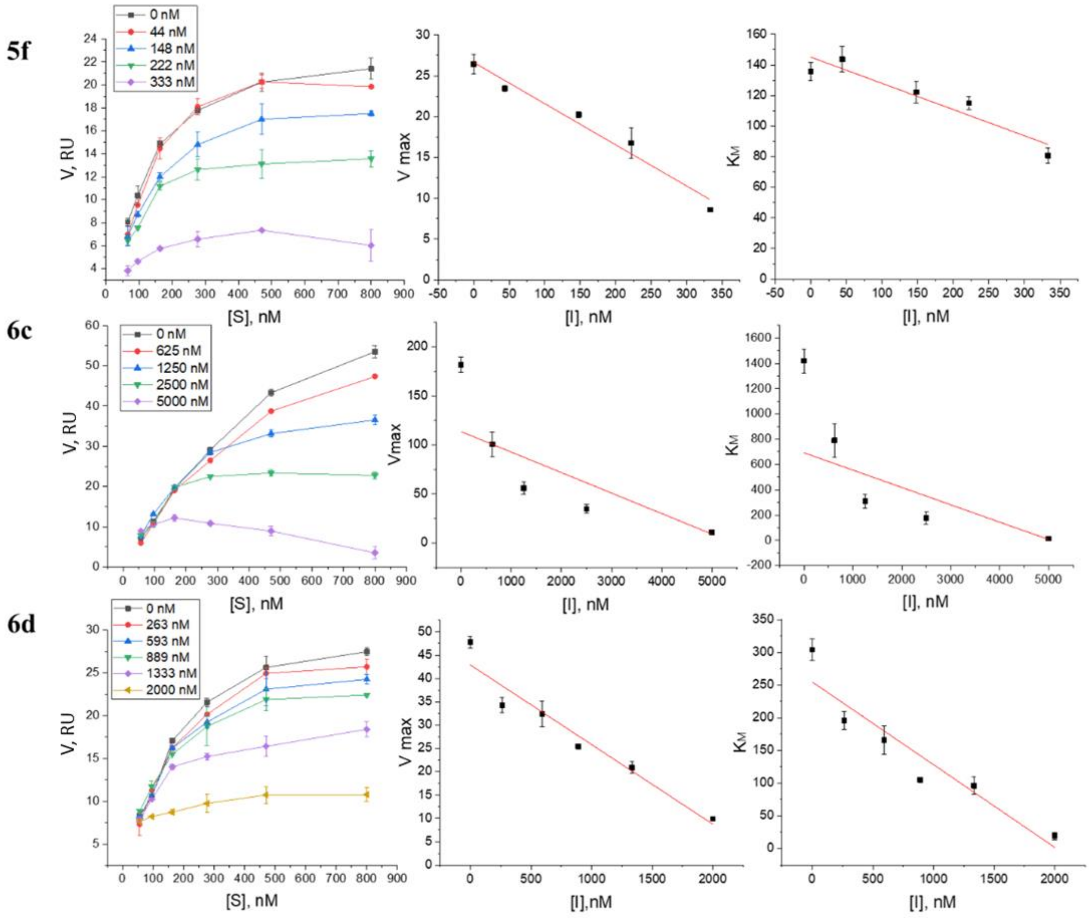

Figure S7. Tdp1 type of inhibition for the most effective compounds. On the left are the graphs of the Tdp1 reaction rate on the substrate concentration in the presence of compounds **5f**, **6c** and **6d**. On the right are the graphs of Michaelis constant K_M and maximum reaction rate V_{max} on Tdp1 inhibitor concentration. There is a proportional decrease in V_{max} and K_M, which is typical for an uncompetitive type of inhibition.

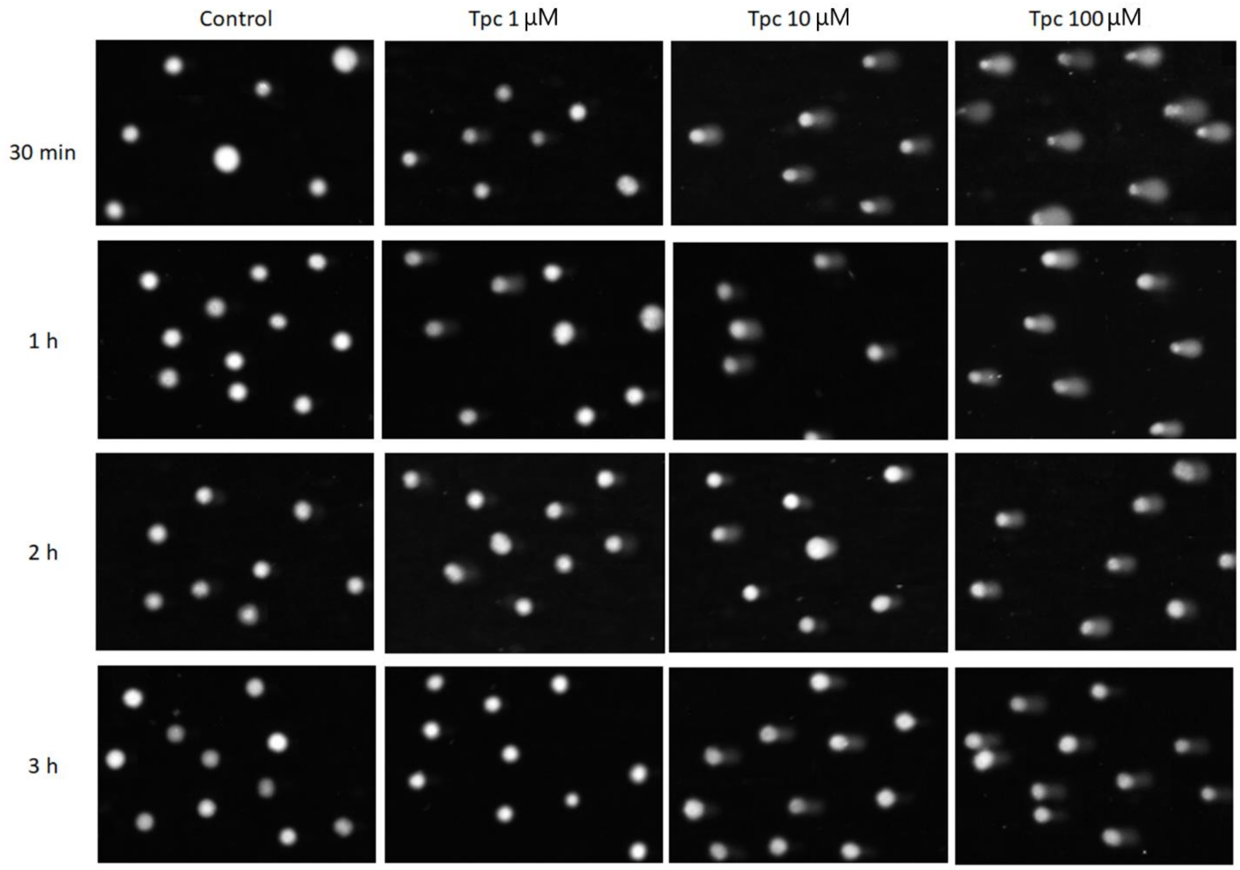

Figure S8. The pictures of cells treated with topotecan, demonstrating the accumulation of DNA damage depending on time.

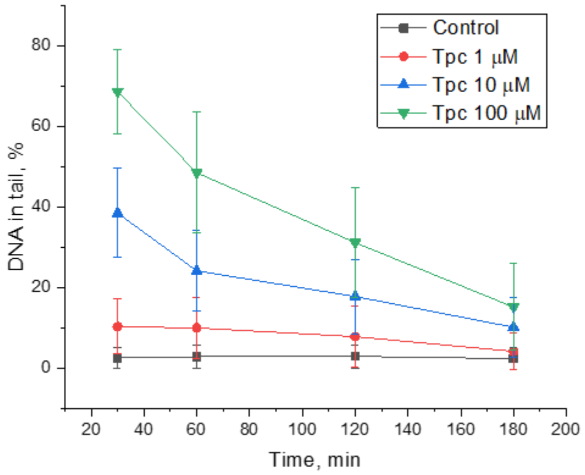

Figure S9. Kinetic curves of generation of DNA damage induced by topotecan. It can be observed, that topotecan caused the greatest amount of DNA damage after 30 minutes treatment, and further the repair systems began to recover DNA damages and as a result, after 3 hours most of them have been eliminated.

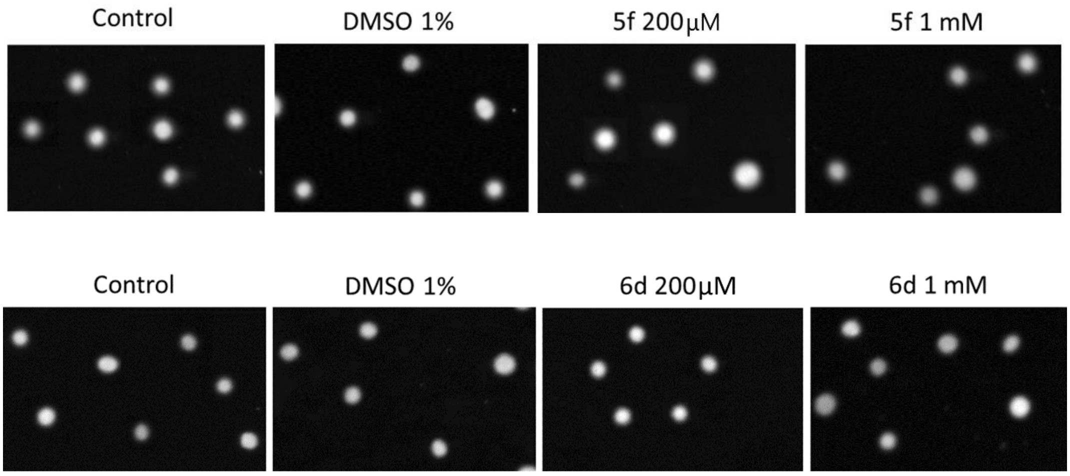

Figure S10. The pictures of control cells (untreated), cells treated with 1% DMSO and leader compounds. There are no tails under the treatments, which indicates the absence of compounds influence on the DNA damage level.

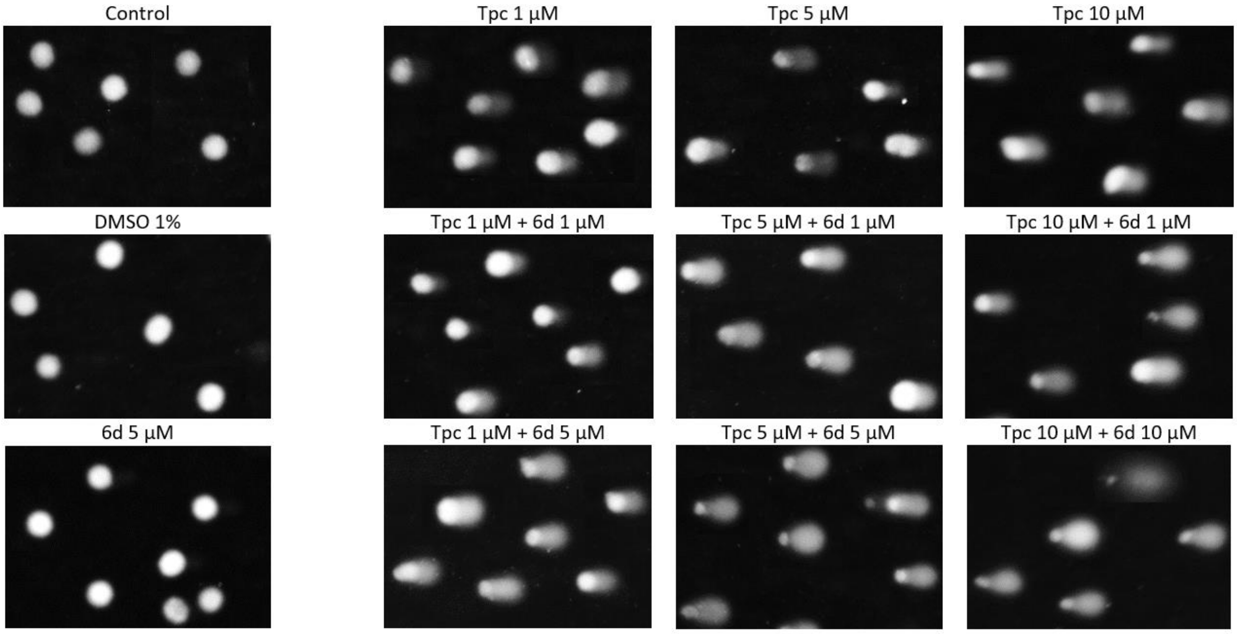

Figure S11. The pictures of cells treated by combination of several topotecan concentrations (1, 5 and 10 μM) and few concentrations of compound **6d** (1, 5 and 10 μM).

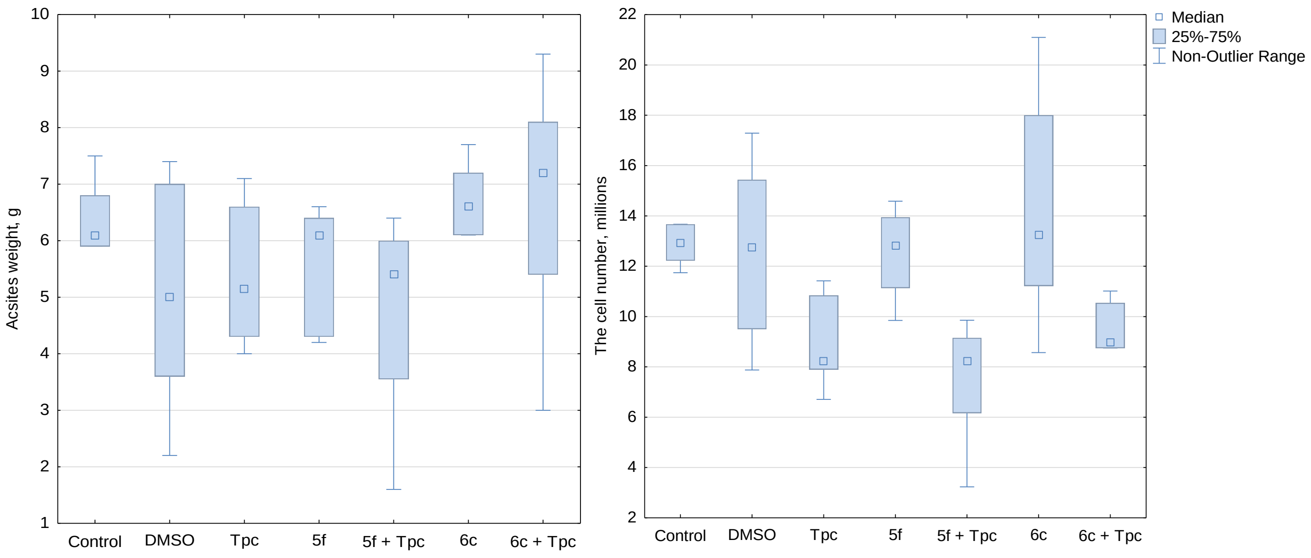

Figure S12. Investigation of the influence of compounds **5f** and **6c** on the antitumor effect of topotecan *in vivo* on a Krebs-2 carcinoma model. On the left are data on the weight of ascites, grams; on the right, the number of tumor cells in ascites, millions.
